# World on Fire

**DOI:** 10.1371/journal.pbio.0020054

**Published:** 2004-02-17

**Authors:** Steve Bunk

## Abstract

Fires are increasing in severity and incidence around the globe and affect many different ecosystems; will the new generation of fire science tools help managers retain biodiversity?

From Bob Clark's snug office in Boise, Idaho, where he manages the United States government's Joint Fire Science Program (JFSP), he figures his computer provides fingertip reach to just about everybody who's anybody in wildfire research. This points to a primary need of nations worldwide in combating the scourge of recurrent wildfires: tools and technology suited to the job. It's no small order in places as economically, socially, and ecologically varied as, say, Brazil, South Africa, Australia, Indonesia, and the United States, which are among the countries where wildfire creates the greatest havoc.

More than 750,000 acres (303,500 hectares) were burned in southern California alone during last year's wildfires. The 2000 season was one of the country's worst on record, destroying 8.4 million acres (3.4 million hectares), more than double the decade's 10-year average. Australia's summer months around the turn of 2002–2003 brought perhaps the worst drought in a century to the populous southeast and the biggest fire season for two decades. Mountain forests were extensively burned and more than 500 houses were lost. In 2002, Brazil suffered 217,000 wildfires, a number that is almost certainly too low because remote imaging cannot detect many fires under the forest canopy. In Indonesia, wildfires that burned for months during 1997–1998 were later estimated to have released the equivalent to 13%–40% of annual global carbon emissions from fossil fuels, inflicting smoke-related ailments on thousands.

Where wildfire is concerned, the many differences between such countries can perhaps be pinned down to two essentials. The first is whether a blaze occurs in temperate or tropical forest, and the second is whether the nation is developed or developing.

“The science can be rock solid, but it can only go so far before social, economic, and political pressures take over,” Clark says. “That's what a forest service manager's job is, picking the best option based on all those considerations.”

Unfortunately, having science-based options that are applicable to local conditions is largely a luxury for developed countries. Managers there can choose to let a fire burn under hopefully contained conditions, a policy known in the United States as “wildland fire use.” They can set experimental crown fires to study their effects, as was done recently in"journal" Canada (Figure 1). And they can take preemptive measures, such as reducing fuel in the forest to lower fire hazard.

**Figure 1:**
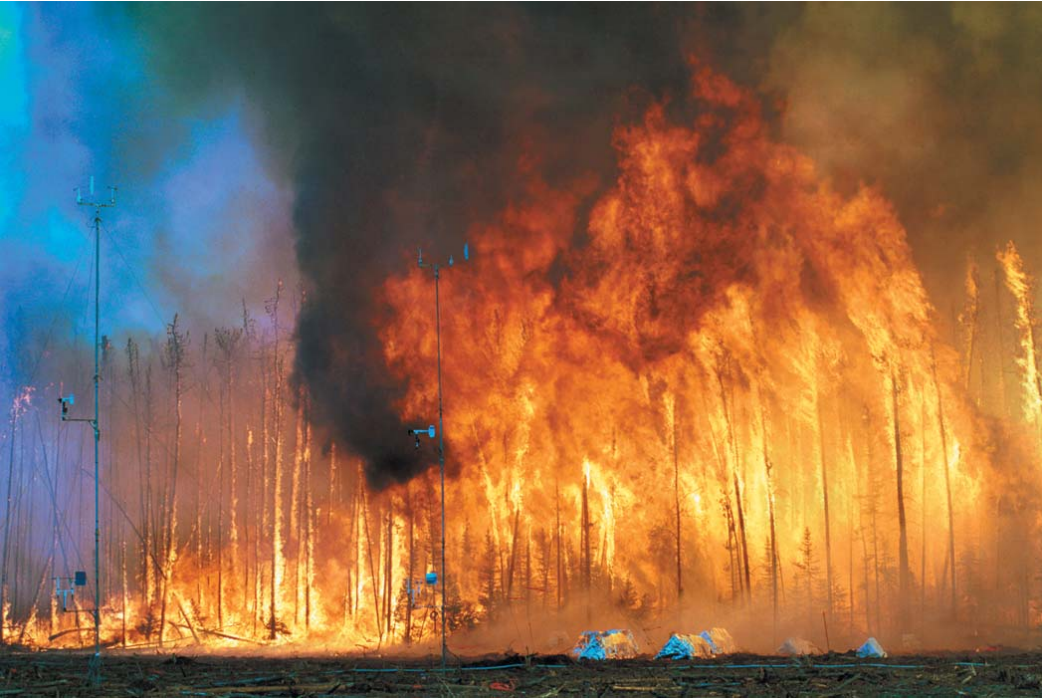
Northwest Crown Fire Experiment (Photograph used by permission of the USDA Forest Service.)

The two main fuel-reduction methods are mechanical removal of combustible materials and controlled or “prescribed” burning (Figure 2). During Bill Clinton's administration, prescribed burns were encouraged in protected areas, but thinning was allowed only for trees with trunks of nine inches (22.8 cm) in diameter or less. Under George W. Bush, prescribed burning remains a choice, but the United States Department of Agriculture's (USDA) Forest Service policy is much more focused on mechanical means. The argument runs that there's been too much concern about removing trees, when what counts most is the enhanced fire-resistance of the thinned habitat.

**Figure 2:**
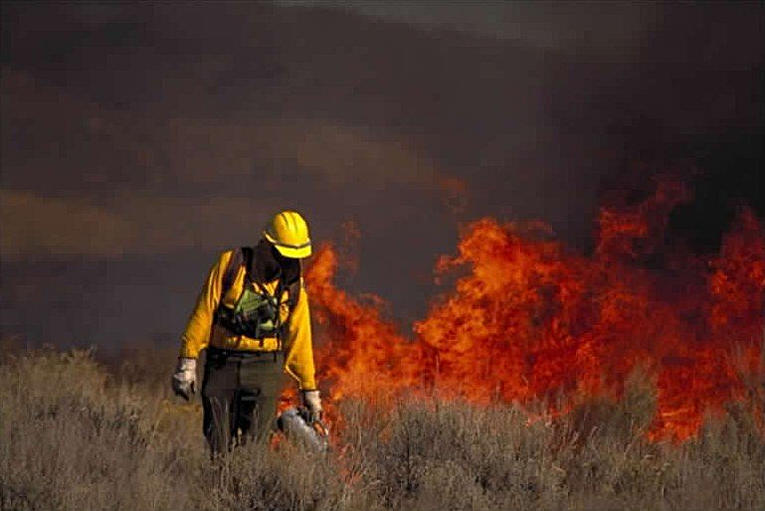
Prescribed Burns in the Intermountain Region of the United States (Photograph used by permission of the USDA Forest Service.)

Fire hazard reduction methods must be tailored to an understanding of fuel characteristics in a given area, says David Peterson of the Forest Service's Pacific Wildland Fire Sciences Laboratory in Seattle, Washington. “There's no uniform way of doing it, partly because, as scientists, we haven't given the management folks any quantitative guidelines.” Working with other ecologists, social scientists, and economists, he's currently producing just such guidelines for the dry interior forests of the Pacific Northwest. “One thing we don't want to do is take choices out of the hands of field managers working at the local level.”

## Forecasting Tools: Models and Simulations

**Figure 3:**
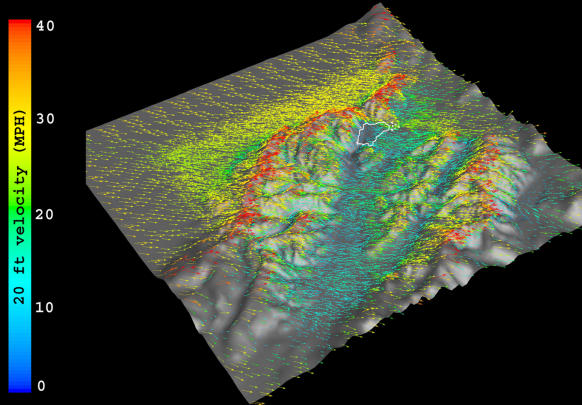
Shaded Surface Images of Areas in Northwestern Montana That Suffered Wildfires during 2003 The winds are from the west at 20 mph/32 kph. The white lines represent fire perimeters. (Image used by permission of the USDA Forest Service, Fire Sciences Laboratory.)

For those choices to be meaningful, managers need reliable information on the risk of wildfire outbreaks and on the future behavior of existing fires. This requires models and simulations that incorporate climatic conditions, particularly wind (Figure 3). At the Forest Service's Fire Sciences Laboratory in Missoula, Montana, researchers have created a “gridded wind” tool based on the engineering discipline of computational fluid dynamics. The program maps wind speed and direction using a digital elevation model, which is a grid of elevation points every 30–100 feet (9–30.5 meters) over a terrain 10–40 square miles (25.9–103.6 square kilometers) in size. This map forms the floor of a box extending up to five miles (eight kilometers) high, which is subdivided into a million or more cubes. Wind flow from either real observations or estimates can be entered into the software, and the layer of cubes nearest the grid floor is used to create surface wind maps at resolutions of every 100 meters (109 yards) or less. In contrast, the usual resolution of weather forecasts is 12 kilometers (7.5 miles), down to 4 kilometers (2.5 miles) in some urban areas.

“Two or three years ago, we couldn't have done this simulation on a single-processor laptop,” says one of its developers, physical engineer Bret Butler. “It would have taken two or three days. Now we can do it in a matter of hours.”

The ability of these maps to show varying wind flow in valleys, at midslope and on ridgetops, is just the beginning. The next step is to feed these data into models that predict wildfire spread. Butler and colleagues have coupled their gridded wind technology to a fire growth model and tested it against the actual spread of several wildfires, including in Southern California last summer. Maps of actual and predicted surface winds showed strong similarities, encouraging Butler to foresee an ideal scenario in which fire fighting teams enter wind flow data online or by telephone to a central base where gridded wind maps and fire growth simulations are generated within hours, before operational decisions are made.

Yet he admits that challenges remain, including the current inability of fire behavior simulation to account for diurnal winds in addition to cold front-driven flow. In mountainous terrain, for example, winds often move up-canyon in the morning and down-canyon in the evening. Moreover, the effect of vegetation on wind is not yet included in such models.

Those issues and others are being addressed by researchers working on improvements to the regional weather forecasts of so-called mesoscale models. At the Forest Service's Rocky Mountain Research Center in Fort Collins, Colorado, meteorologist Karl Zeller and colleagues are contributing calculations of biological processes to mesoscale weather models. Their algorithms not only can account for diurnal winds but can predict the effects on local weather when vegetation takes in carbon dioxide and releases water vapor. This process can produce different fluxes of carbon dioxide drawn into the canopy and water vapor coming out, depending largely on the type of vegetation and its canopy density.

Zeller's group has analyzed current mesoscale forecasts in the Rocky Mountains and found that in the daytime, they often are too hot in the high country and too cold in the plains. Water vapor estimates are too low in the mountains and too high in the plains, which Zeller thinks is because the models feed off soil moisture estimates, not off vegetation. In coupling his team's new biophysical interface to gridded wind and mesoscale forecast models, Zeller says “point forecasts” are being developed that can focus on a prescribed burn area or even a single house.

## Wildfire and Species Diversity

Fitting the appropriate mix of strategies to a given situation is an issue that has also received close attention in Australia. After the bushfires of 2002–2003, media commentators called for increased “hazard reduction burning” in national parks, prompting ecologists around the country to distribute a joint statement declaring that such a strategy would not further reduce bushfire risk, but would actually threaten biodiversity. Australian species are often well-adapted to fire, and researchers have learned that different fire regimes—meaning the type of fire, its intensity, severity, extent, season, and frequency—favor different species (Box 1). In the southeast of Australia, prescribed burns of high frequency and low intensity can alter the habitat in ways that therefore threaten survival of numerous plant and animal species.

“A generic problem or conundrum seems to be that species which do not prosper under relatively frequent fires can be found in most fire-prone environments,” notes Ross Bradstock, principal research scientist in the New South Wales Department of Environment and Conservation. He says it's very difficult to determine how human interventions in various habitats can foster the coexistence of species that have different fire regime requirements.

## Fire Suppression and Tropical Forests

**Figure 4:**
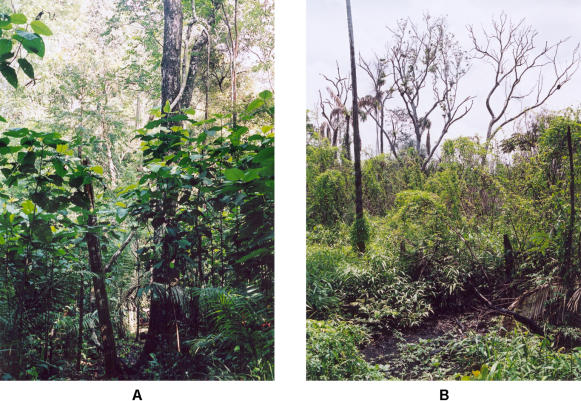
Forest Regeneration (A) Dense understory regeneration three years after a low-intensity fire. (B) The almost total loss of live, above-ground biomass six months after a forest burnt for the third time in 15 years. (Photographs by Jos Barlow; used by permission.)

As tough as such questions are to answer in developed countries, they pale compared to the problems of tropical forest wildfire researchers and managers in developing countries. In these countries, a destructive cycle of human behavior begins with land-clearing and burning for farming, logging, mining, road-building, and other uses that open gaps in the rainforest's canopy cover. This lets in sunlight and air, reducing the forest's ability to smother fire by trapping moisture, and it encourages the growth of smaller, more fire-prone plants. The first wildfires that occur are bad, but successive ones can eventually transform tropical forest to scrub savanna (Figure 4). Of course, the remaining forest is thereby broken into fragments that continue to suffer incursions at their edges, as the cycle continues.

In a recent paper in Science, Michigan State University Amazon expert Mark Cochrane pointed out that prescribed burning is ineffective in tropical forests, because the collateral damage outweighs any benefits. Indeed, tools and technologies employed in temperate conditions can seldom be applied usefully to tropical forests without significant alterations.

“One of the main issues in fire science is that the U.S. has no capacity to develop new tools,” charges Ernesto Alvarado, a research scientist at the University of Washington in Seattle. He's been working for several years with United States Forest Service and Brazilian scientists on field studies in Mata Grosso, the southernmost state of the Brazilian Amazon. He says that fire prediction simulations developed decades ago have not yet been replaced by ones that account for tropical wildfire extremes, including either large-scale crown fires or surface fires, which often reach only 10 centimeters (3.9 inches) in height and move slowly but can burn for weeks and kill many trees.

 Fire behavior models don't work for tropical surface fires because the physics are different from those in temperate forests, he explains. A slow wind generated from the unburned forest blows toward the fire, forcing the small flames to advance against, rather than with, the wind. Another difference is that the fuel is mostly leaf litter, not conifer needles or sticks.

Alvarado and colleagues light experimental fires in clear-cuts to determine factors limiting ignition and spread. Such experimental work is rare in tropical forests, where observation and description still predominate. But the team also monitors surface wildfires, measuring fire length, spread, and heat release.

“We're trying to find applications that people can use to control fires or to explain implications of fire policy,” he says. Most wildfires originate from deliberately set burns. For example, many farmers still clear land by the ancient method of slash-and-burn, in which forest is chopped, left to dry, and then burned. These farmers are now banned by Brazilian federal law from burning at the height of the dry season, mid-July to mid-September. They cut in May, but if the rains come early in September, they can't burn after the ban ends and must wait until the next season, with nowhere to grow their crops in the meantime. Alvarado thinks a more flexible burning schedule is a solution.

The challenge is to pass on technological understanding to decision-makers. For example, even ranchers in Mata Grosso's economic elite usually haven't heard of fire management techniques, says Amazonian ecologist Carlos Peres at the University of East Anglia in the United Kingdom. Educational projects from nongovernmental organizations have helped to turn some farmers away from heavy reliance on slash-and-burn techniques, but fire suppression information remains to be distributed on the frontiers.

“What we really need are very large areas of primary forest that effectively serve as fire breaks,” he says. Conservation plans have been made by the federal government in collaboration with international agencies, but implementation remains a question, particularly given the high level of economic pressure from multinational resource developers eager to enter the Amazon. Major roads through the jungle are also on the drawing board. “Different categories of conservation units can be gazetted on paper, but in practice they're a long way from working. Someone draws lines on a map high in an office in Brasilia, but when you go out to that place in the forest, no one knows it's a conservation zone.”

## Fire Prevention: Developing the Technology

Information transfer faces similar barriers in much of Southeast Asia, as Canadian forestry researchers discovered during a five-year project (now winding up) to create a computerized early warning tool for wildfire outbreaks. The program was instigated after the 1997–1998 fires created a regional haze hazard, largely because of peat deposits up to 20 meters (21.8 yards) thick that had become susceptible to burning in swampy forests drained and cleared for development.

Michael Brady, who managed the Canadian project in Jakarta, points out that headmen in remote communities are still likely to believe that wildfires start spontaneously, by grasses rubbing together or even by magic. A fire scientist whose doctorate is in tropical forest peat dynamics, Brady sees the project as a medium to strengthen regional fire ecology in general. “In some ways, that's more important to me than the tool itself.”

The tool is a variation of the Fire Danger Rating System used in Canada and, with various permutations, in many other countries. The Canadian system has two components, one for indexing fire weather and another predicting fire behavior. The weather component models moisture input and output in fuels generically classed as fine, moderate, and heavy. Brady and Indonesian university scientists grouped grasslands in the fine fuel category, fallen leaves and litter as medium, and peat and woody materials as heavy. They spent three years calibrating these fuels to local weather conditions, examining moisture dynamics and performing ignition tests. In developed countries, fuels are further specified in numerous classes for fire behavior prediction, but that requires decades of field work. Brady's team concentrated instead on helping key agencies in seven Southeast Asian countries, especially Indonesia and Malaysia, to obtain and use the appropriate computing tools.

Brady doesn't expect immediate results in terms of reducing acreage burned. “Canada and the U.S. still have huge fire problems after working on it for a century.” But he does hope for a change of thinking, away from a current fascination in the region with satellite imaging of “hot spots” where fires are likely to be occurring. Fire danger rating concentrates on where fires are most likely to begin. “It allows you to add prevention into your management program.”

## Beyond Prevention

In South Africa, “retention” is a conservation buzzword referring to strategies that, in a sense, go beyond prevention of problems. What ecologists hope to retain is biodiversity in the midst of changes that can't be stopped, and their methods are producing major repercussions throughout government. The work is centered on the Cape Floristic Region of Africa's southwestern tip. Almost 90,000 square kilometers (34,750 square miles) in area, it's the world's smallest floral kingdom. A conservation plan was launched in 1998 that has drawn cooperation from tourism, mining, water use, agricultural, and land use planning groups.

The project has the ambitious goal of protecting not only the usual biodiversity patterns of conservation areas but also the spatial components of evolutionary processes that enable species to adapt to potentially harmful changes. This entails a complex effort to determine which parts of developed and undeveloped lands are most necessary to such processes, including rivers, sand movement corridors, gradients from uplands to coastal lowlands, and major wilderness areas. University of Port Elizabeth botanist Richard Cowling, one of the scheme's principal architects, estimates that it might require 60%–70% of the region's landscape.

As in Australia, fires are important to the Cape's biodiversity, but too-frequent burns are a problem. Cowling thinks that by consolidating mountainous megawilderness under the project's plan and protecting spatial transitions between fire-prone areas and those that resist fire, managers could move toward allowance of natural fire regimes. The current problem, he says, is that protected areas usually stop short of the transition to semidesert areas that are privately owned. When fire spreads from public to private land, the government often gets sued. Under the evolving Cape plan, landowners will sit on governing boards, and property that they contract for conservation will be tax-exempt.

The Cape plan has attracted millions of dollars in support from the World Bank and other international sources, but Cowling regards that achievement as much less important than the progress made in gaining support from various interest groups. “The key issue is the extent to which you can get biodiversity concerns mainstreamed to other sectors,” he says. Threats to habitat retention, not least of which is wildfire, endanger every species. “It's about making people realize that biodiversity is the basis upon which all other things will succeed.”

##

**Box 1. Fire-Adapted Species**

Plants and animals of many countries evolved for millennia with wildfire as a natural occurrence, but when human interventions increase the frequency of fire, species suffer. African fire lilies and Australian “grass trees” are among plants that are stimulated to flower by smoke constituents such as ethylene. Plant seeds in fire-prone landscapes of Australia and South Africa often require fire to stimulate their germination, but it can take more than a decade for new seed banks to mature in some species. If a second fire arrives before then, the species could die out.

Animals can be similarly affected. For example, a threatened marsupial called the potoroo is capable of surviving a high-intensity wildfire, but cannot tolerate the habitat changes caused by frequent, low-intensity fires. Likewise, some species of Australian honeyeaters are threatened with extinction because too-frequent fires have changed the proportion of mature and immature nectar plants. On the other hand, ecosystems can also be transformed by fire suppression. In southern Africa, decades of such activity have encouraged forests to replace grasslands. Yet the lovely marsh rose almost disappeared from the Cape before land managers realized that fire suppression was preventing its seeds from germinating. In such ways, biodiversity must find its place among the goals and tradeoffs of human intervention.

## Abbreviations

JFSP: Joint Fire Science Program
USDA: United States Department of Agriculture
